# Small Bowel Hamartoma: A Huge Diverticulum of Small Bowel

**DOI:** 10.1155/2013/970457

**Published:** 2013-12-18

**Authors:** Hamdi Ebdewi, Amar M. Eltweri, Yahya Salama, Neshtman Gorgees, Leena Naidu, David J. Bowrey

**Affiliations:** ^1^Department of General Surgery, Kettering General Hospital, Kettering NN16 8UZ, UK; ^2^Department of UGI Surgery, University Hospital of Leicester, Leicester Royal Infirmary, Leicester LE1 5WW, UK; ^3^Department of Histopathology, Kettering General Hospital, Kettering NN16 8UZ, UK; ^4^Department of Radiology, Kettering General Hospital, Kettering NN16 8UZ, UK

## Abstract

A-20-year old male, with no significant medical history, presented with clinical features mimicking a perforated acute appendicitis. Because of features of peritonitis, a laparotomy was performed which showed a segment of small bowel with multiple large diverticula and mesenteric cysts. A segmental small bowel resection was performed. The patient made an uneventful recovery from surgery. Histology revealed features of a small bowel hamartoma.

## 1. Background

The rarity of this lesion made the histological diagnosis a debatable issue. Histologically, the unusual appearance and the presence of the mixed vascular, lymphatic, smooth muscular, and adipose tissue components in the identified lesion meets the principles implied in the concept of hamartoma. Hence, we present a rare case report of small intestine (ileum) hamartoma.

## 2. Case Presentation

A 20-year-old male was admitted via the Emergency Department on the Acute General Surgical Intake with a 24-hour history of right iliac fossa pain, in association with vomiting. His only medical history was of acne vulgaris for which he received Lymecycline 408 mg OD. He had experienced similar abdominal pain for a seven-day period, one month earlier. At that time, laboratory investigations and plain radiology were unremarkable. The patient was offered laparoscopy and appendicectomy, declined intervention, and was self-discharged against medical advice as he felt improvement. Examination findings on the readmission comprised a fever (temperature 39.6°C), pulse rate of 93 beats per minute, a respiratory rate of 19/minute, oxygen saturation of 96% on air, and normal Glasgow Coma Score. Palpation of the abdomen revealed diffuse tenderness throughout, with generalised guarding with no abdominal distension, and bowel sounds were absent.

## 3. Investigations

Laboratory investigations showed haemoglobin of 16.7 g/dL, white cell count of 13.6, normal renal function, and normal C-reactive protein. A plain abdominal radiograph was normal. In view of the degree of tenderness, a contrast-enhanced CT scan of the abdomen and pelvis was obtained. This revealed what looks like a 50 cm multiseptated collection, occupying much of the abdominal and pelvic cavities. The collection contained pockets of air. There was a small quantity of free fluid around the liver and in both paracolic gutters. The appendix was not visualised.

## 4. Management

Intravenous fluids, broad spectrum antibacterials, and proton pump inhibitors were administered. The patient was catheterised and taken for emergency exploratory laparotomy.

## 5. Outcome and Followup

**Figure 1:**
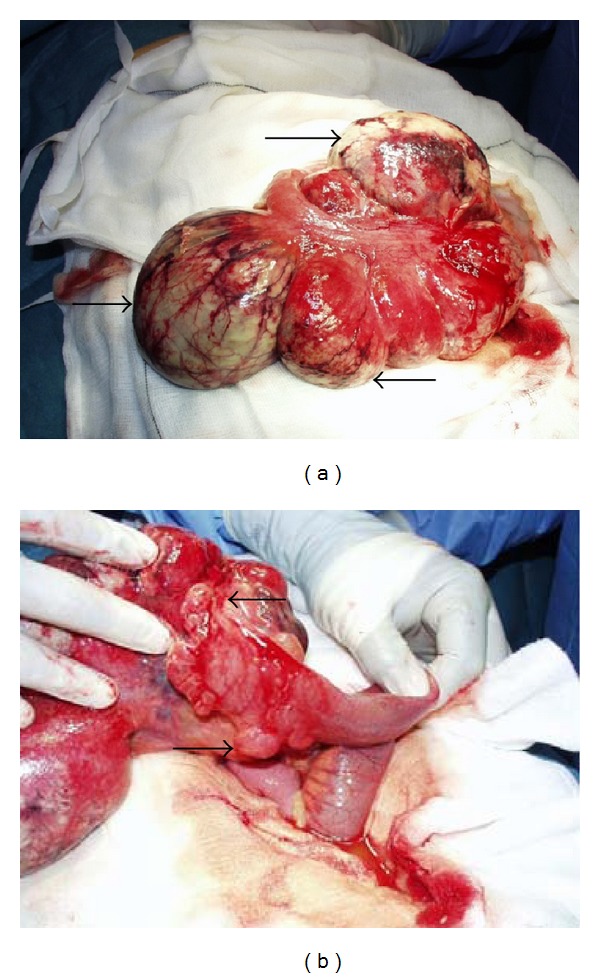
Macroscopic appearance of the ileal diverticula (a) (arrows) and multiple mesenteric cysts (b) (arrows).

**Figure 2:**
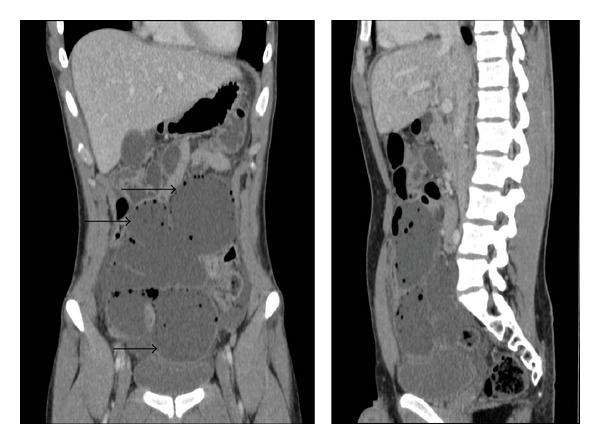
CT scan appearance of the small bowel hamartoma (large diverticula arrows).

At laparotomy, a 20 cm abnormal segment of small intestine (ileum) was identified; this gave a false appearance of 50 cm collection on the CT scan images (Figures 1 and 2) that was characterised by multiple diverticula and mesenteric cysts. A small bowel resection with primary anastomosis was performed. The patient made an uneventful recovery and was discharged home on the 7th postoperative day.

Interpretation of the resection specimen histology proved challenging. Macroscopically, the resected 25 cm segment of small bowel contained two thin-walled cysts located on the mesenteric border, one 15 by 14 cm and the other 11 by 1 cm. There was evidence of perforation and a purulent serosal reaction. On sectioning, both lesions showed “honeycombing” with numerous cystic loculi, with their size ranging between 5 and 20 mm. The small bowel mucosa had a granular, thickened, and ulcerated appearance.

Microscopically, the cystic (diverticula) lesions were characterised by numerous mural and subserosal dilated vascular spaces, lined by endothelium surrounded by a layer of smooth muscle fibers. Some of the spaces contained proteinaceous lymph-like material, and others contained blood. The small intestine showed focal ulceration. The lamina propria were expanded with numerous small lymphatic channels. There was evidence of perforation and an acute suppurative serositis. There was no evidence of granulomata or malignancy.

The differential diagnosis lay between cystic lymphangioma, angiomyolipoma, and hamartoma. The consensus opinion was that the lesion was a hamartoma.

The patient was followed up in clinic six months postoperatively with a repeat normal CT scan abdomen and pelvis and was discharged without any further followup.

## 6. Discussion

Hamartoma is very rare benign condition associated with an abnormal location and arrangement of tissues normally found in small intestine [1]. Diagnosis is usually made by histological examination. The most common presenting symptoms are of intestinal obstruction due to either intussusception or stricture. The previously reported types of hamartomas of the ileal part of small intestine were neuromuscular and vascular hamartoma (NMVH), neuromesenchymal hamartoma (NMH), myoepithelial hamartoma (MEH), and Cowden hamartomatous syndrome Table 1 summarises the reported cases of terminal ileum hamartomas.

In 1940, Clark, used the term myoepithelial hamartoma (MEH) to describe the submucosal masses of epithelial glandular cells and smooth muscles were found in eight cases [2]. Myoepithelial hamartoma is a very rare benign tumour-like lesion of the gastrointestinal tract and the small intestine is the second common location and usually in the periampullary region, a pancreaticoduodenectomy is often performed in this situation, as it is difficult to distinguish it from carcinoma [3], and MEH usually presents with intussusception [4]. Fernando and McGovern, in 1982, were the first to present two cases of NMVH.

Nakayama et al. present a case of Cowden hamartomatous syndrome complaining of GIT bleeding and melena. It was identified as having multiple GIT hamartomas with arteriovenous malformation as a source of the bleeding, and the histopathology of the resected jejunal and ileal specimen showed the presence of adipose tissue and ectatic lymphatic vessels [5].

**Figure 3:**
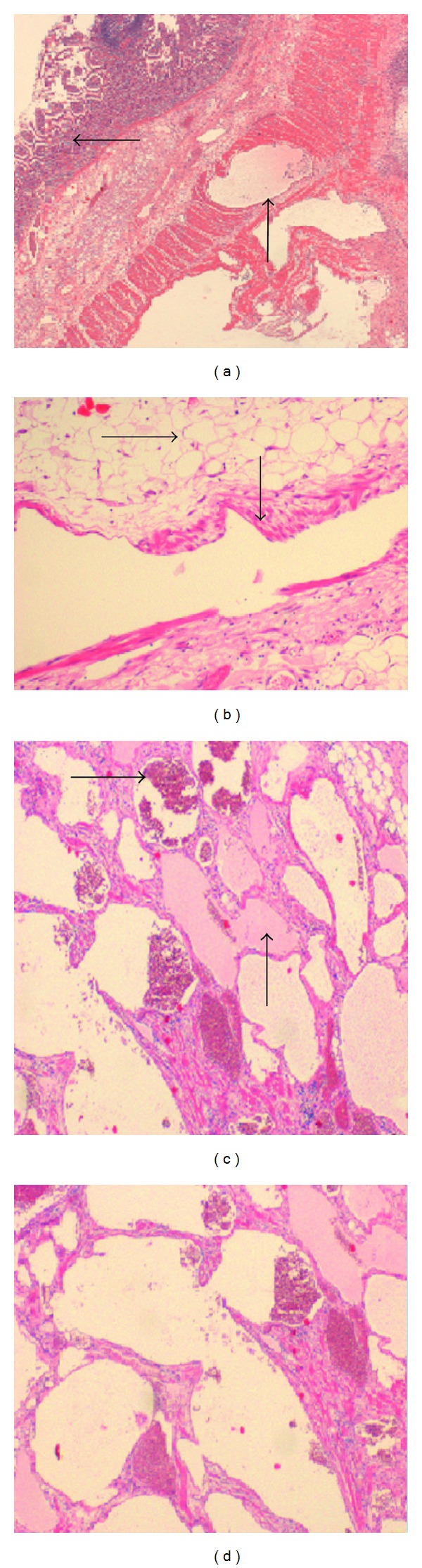
Histology images of the reported small bowel hamartoma. (a) Normal small intestinal mucosa top arrow and dilated thin-walled vascular spaces in the muscularis propria bottom arrow. (b) High power viewing of the vascular lesion in which a dilated, vein-like blood vessel with some smooth muscle fibres in the wall bottom arrow and adipose tissue top arrow. (c) A closer view of the lesion, with many closely packed vascular and lymphatic spaces within the bowel wall, contains blood (top arrow) and proteinaceous lymph-like material (bottom arrow), respectively. (d) Magnified view of the aggregated abnormal vascular “some have prominent muscular wall” and lymphatic spaces.

To our knowledge, we present the first case of small bowel hamartoma in young male manifested with lower abdominal pain, and CT scan showed massively dilated small bowel mimicking large intra-abdominal collection (Figure 2). Intraoperatively, the affected segment of terminal ileum showed large diverticulum-like lesions with no obvious obstructive points (Figure 1). The diagnosis was challenging for the histopathologists and showed a mixture of smooth muscles, adipose tissue, vascular and lymphatic vessels and some of which filled with clotted blood or proteinaceous lymph-like material, respectively (Figure 3).

## 7. Learning Points/Take Home Messages

Small intestine (ileum) hamartoma is a rare benign entity, but like other small bowel tumours, it presents with features of obstruction and is managed by small bowel resection.

## Figures and Tables

**Table 1:** Summaries of the reported small intestine (ileal) hamartomas with the macro- and microscopic appearances.

Author	Hamartoma	NOC reported	Age and sex	Presentation/symptoms	Treat.	Macroscopic appearance	Microscopic appearance
Fernando and McGovern (1982) [6]	NMVH	2	30 y/F	Chronic GIT bleeding	SR	Stricture	SM, NF, and VC
			36 y/F	Abdo. pain, vomiting, and constipation	SR	Stricture	
Smith et al. (1986) [1]	NMVH	1	50 y/F	Ch. abdo. pain, vomiting, and abdo. mass	SR	Stricture	SM, NF, and VC
Shepherd and Jass (1987) [7]	NMVH	4	34 y/F	Ch. abdo. pain and D&V.	SR	Stricture	SM, NF, and VC
			58 y/M	Ch. abdo. pain and diarrhea		Stricture	
			73 y/F	Ch. abdo. pain		Stricture	
			63 y/F	Colicy abdo. pain		Stricture	
Kwasnik et al. (1989) [8]	NMVH	1	91 y/M	GIT bleeding and IO symptoms	SR	Stricture and mass	SM, ganglia, fib. T., lymph., and VC
Salas et al. (1990) [9]	NMH	1	55 y/F	Ch. abdo. pain, dist., and diarrhea	SR	Stricture	SM, fib. and adipose T., NF, and VC
Gonzalvez et al. (1995) [10]	MEH	1	2 y/M	Vomiting and GIT bleeding	SR	Intussusception	SM and GE
Yamagami et al. (1997) [11]	MEH	1	4 m/M	Abdo. pain and vomiting	SR	Intussusception	GE and SM
Di Benedetto et al. (1998) [12]	IH	1	12 y/M	Abdo. pain, vomiting, and rectal bleeding	SR	Intraluminal polypoid mass	GE and SM
Cortina et al. (1999) [13]	NMVH	2	73 y/M	IO symptoms	SR	Stricture	SM, NF, VC, collagen, and ganglia
			76 y/M	IO symptoms		Stricture	
Zolota et al. (2000) [14]	NMVH	1	40 y/M	Abdo. pain and diarrhoea followed by IO	SR	Stricture	SM, NF, and VC
de Sanctis et al. (2001) [15]	NMVH	1	76 y/F	IO symptoms	SR	Stricture	SM, NF, VC, and fibrous tissues
Ikegami et al. (2006) [4]	MEH	1	5 m/F	Vomiting	SR	Intussusception	GE and SM
Lin et al. (2011) [16]	Hamartoma	1	13 y/M	Abdo. pain, constipation	SR	Intussusception	Adipose tissues and VC
Krishnamurthy et al. (2010) [17]	NMVH	1	32 y/M	IO symptoms	SR	Intussusception	SM, NF, VC, and lymphoplasmacytic infiltrates

NOC: number of cases, Treat.: treatment, NMVH: neuromuscular and vascular hamartoma, MEH: myoepithelial hamartoma, NMH: neuromesenchymal hamartoma, IH: intraluminal hamartoma, GIT: gastrointestinal tract, Ch.: chronic, abdo.: abdominal, D&V: diarrhoea and vomiting, dist.: distension, IO: intestinal obstruction, SR: surgical resection, SM: smooth muscles, NF: nerve fibres, VC: vascular channels, lymph.: lymphatics, GE: glandular epithelium, and fib. T.: fibrous tissues.
